# Editorial: Cardiopulmonary exercise testing in chronic diseases

**DOI:** 10.3389/fspor.2025.1598498

**Published:** 2025-04-07

**Authors:** Aikaterini Gakidi, Serafeim-Chrysovalantis Kotoulas, Athanasia Pataka, Georgia Pitsiou, Afroditi Boutou

**Affiliations:** ^1^ Department of Respiratory Medicine, Hippokration General Hospital of Thessaloniki, Thessaloniki, Greece; ^2^ Adult Intensive Care Unit, Hippokration General Hospital of Thessaloniki, Thessaloniki, Greece; ^3^ Department of Respiratory Failure, Aristotle University of Thessaloniki, Thessaloniki, Greece

**Keywords:** cardiopulmonary exercise testing, pulmonary arterial hypertension, obstructive sleep apnea, prehabilitation, heart failure

**Editorial on the Research Topic**
Cardiopulmonary exercise testing in chronic diseases

Cardiopulmonary exercise testing (CPET) is a noninvasive tool that allows the dynamic evaluation of the body's responses to exercise, involving the pulmonary, cardiovascular, hematopoietic, neuropsychological, and skeletal muscle systems, which are not adequately reflected through the measurement of individual organ system function. CPET plays a crucial role in distinguishing between cardiac, pulmonary, or deconditioning-related undiagnosed exercise intolerance and dyspnea, as well as assessing functional capacity and disease severity in cardiovascular and pulmonary diseases. CPET is also widely used in prescribing exercise programs and evaluating the effectiveness of rehabilitation interventions. Furthermore, it is useful for preoperative risk stratification for major surgeries (1, 2).

The role of exercise in managing and improving cardiopulmonary health continues to gain prominence, with growing evidence supporting its benefits across a range of conditions. Exercise intolerance refers to a diminished capacity to perform physical activities or exercise that would typically be expected for a person's age and size (3) and is linked to the severity of multiple diseases, such as chronic obstructive pulmonary disease (4), interstitial lung diseases (5), pulmonary hypertension (PH) (6) and heart failure (7). This issue brings together four insightful articles that explore different aspects of exercise interventions and cardiopulmonary testing, providing valuable perspectives both for researchers and clinicians.

Coulis et al. presented in a narrative review the role of CPET in Pulmonary Arterial Hypertension (PAH) and Chronic Thromboembolic Pulmonary Hypertension (CTEPH). CPET is a valuable tool to evaluate functional limitation and exertional dyspnea in PAH and CTEPH patients; by recording numerous breath-by-breath parameters it offers valuable information regarding cardiac function and pulmonary gas exchange abnormalities. In both PAH and CTEPH increased pulmonary vascular resistance leads to reduced oxygen delivery. Key abnormalities detected by CPET are regional hypoperfusion relatively to ventilation (lower PETCO_2_), impaired stroke volume response, high ventilatory equivalent for carbon dioxide (VE/VCO_2_) and low peak O_2_ uptake (peak VO_2_). Apart from differential diagnosis of PH from other causes of dyspnea, CPET is useful for risk stratification of PAH/CTEPH patients, monitoring their disease progression and treatment response, as well as assessing their prognosis (e.g., reduced peak VO₂ and increased VE/VCO₂ slope are associated with poor outcomes in PAH). More studies are needed to refine CPET-based diagnostic criteria for PAH/CTEPH. Identifying abnormal responses to exercise with the use of CPET can also be used for guiding personalized treatment strategies and exercise protocols.

Additionally, in this issue Wang et al. systematically reviewed the current literature on exercise interventions in heart failure (HF) patients and created an evidence map to visualize research volume and literature gaps on the topic. According to their findings, over 80% of studies concluded that patients in intervention groups outperformed control groups, highlighting the positive impact of exercise in HF. Regarding the type of exercise, mixed-modality training (a combination of aerobic and resistance exercise) gained researchers’ most interest. Current literature predominantly focuses on enhancing exercise capacity, cardiorespiratory function and quality of life and evaluates interventions in patients with HF with reduced ejection fraction. Additionally, this study suggests that future research efforts should be directed toward conducting higher quality primary studies, using a more precise HF classification and broadening outcome measures, such as inflammatory markers. Overall, exercise interventions improve heart failure outcomes, and CPET is essential for measuring and guiding these interventions.

Further expanding on the topic, this issue contains two exercise protocols in clinical populations. With remote healthcare solutions becoming increasingly relevant, Stavrou et al. propose a randomized-controlled trial protocol study in order to evaluate the effectiveness of a 12-week tele-exercise program for individuals with obstructive sleep apnea (OSA). Study population will be patients with OSA and cognitive impairment and will be randomized into two groups; one group will undergo the tele-exercise program and the second will not, while a third group of OSA patients without cognitive impairment will act as a second control group. All participants will not receive continuous positive airway pressure (CPAP) treatment (delayed therapy). Exercise protocol will consist of three 60 min training sessions per week with aerobic and multi-joint strength exercises. Since OSA impacts negatively the cognitive function, researchers hypothesize that this protocol will affect sleep architecture and offer beneficial changes in cognitive function, cerebral oxygenation and CPET parameters. Tele-exercise as complementary to CPAP treatment may improve significantly the quality of life of OSA patients by overcoming limitations, such as economic costs and accessibility of face-to-face rehabilitation programs.

Finally, the second study protocol of the issue explores the concept of prehabilitation exercise in cancer care. Generally, prehabilitation is a wide term including interventions administered prior to surgery to enhance resilience and improve post-treatment recovery, such as physical activity, nutrition support, smoking and alcohol cessation, education and combined interventions (8). More specifically, Chmelova et al. present a single-group interventional feasibility study of an exercise protocol for patients during neoadjuvant therapy with cancer of the upper gastrointestinal tract and rectum that will be conducted at home and monitored telemetrically. All enrolled patients will participate in the protocol consisting of progressively dosed walking, based on each patient's baseline physical activity, and strength exercises. The length of the intervention will depend on the length of oncological treatment. The primary outcome of this study is to assess the feasibility of the exercise protocol by evaluating the proportion of all eligible diagnosed individuals who will agree to participate, the percentage of them that will complete the program, their adherence and any relative adverse events. Researchers also expect changes in CPET parameters, grip strength measured via hand dynamometer, body composition, Borg's perceived exertion scale scores and in health-related quality of life.

In conclusion, by providing valuable insights into cardiopulmonary function, CPET can be used to aim differential diagnoses, stratify risk, guide treatment, optimize rehabilitation strategies and evaluate treatment efficacy of medical and non-medical interventions in chronic diseases. Its ability to assess integrative physiological responses to exercise makes it a powerful tool in modern clinical practice. The editors hope that the articles of this issue will offer new insights that may further enhance clinical decision-making and shape future research around this topic.
